# Genotype Phenotype Correlation in Dent Disease 2 and Review of the Literature: *OCRL* Gene Pleiotropism or Extreme Phenotypic Variability of Lowe Syndrome?

**DOI:** 10.3390/genes12101597

**Published:** 2021-10-11

**Authors:** Lisa Gianesello, Jennifer Arroyo, Dorella Del Prete, Giovanna Priante, Monica Ceol, Peter C. Harris, John C. Lieske, Franca Anglani

**Affiliations:** 1 Kidney Histomorphology and Molecular Biology Laboratory, Nephrology, Dialysis and Transplantation Unit, Department of Medicine-DIMED, University of Padua, 35128 Padua, Italy; lisa.gianesello@unipd.it (L.G.); dorella.delprete@unipd.it (D.D.P.); giovanna.priante@unipd.it (G.P.); monica.ceol@unipd.it (M.C.); 2 Division of Nephrology and Hypertension, Mayo Clinic, Rochester, MN 55905, USA; Arroyo.Jennifer@mayo.edu (J.A.); Harris.Peter@mayo.edu (P.C.H.); Lieske.John@mayo.edu (J.C.L.)

**Keywords:** Dent disease 2, *OCRL* mutations, OCRL domains, genotype–phenotype correlations, Lowe syndrome

## Abstract

Dent disease is a rare X-linked renal tubulopathy due to *CLCN5* and *OCRL* (DD2) mutations. *OCRL* mutations also cause Lowe syndrome (LS) involving the eyes, brain and kidney. DD2 is frequently described as a mild form of LS because some patients may present with extra-renal symptoms (ESs). Since DD2 is a rare disease and there are a low number of reported cases, it is still unclear whether it has a clinical picture distinct from LS. We retrospectively analyzed the phenotype and genotype of our cohort of 35 DD2 males and reviewed all published DD2 cases. We analyzed the distribution of mutations along the *OCRL* gene and evaluated the type and frequency of ES according to the type of mutation and localization in OCRL protein domains. The frequency of patients with at least one ES was 39%. Muscle findings are the most common ES (52%), while ocular findings are less common (11%). Analysis of the distribution of mutations revealed (1) truncating mutations map in the PH and linker domain, while missense mutations map in the 5-phosphatase domain, and only occasionally in the ASH-RhoGAP module; (2) five *OCRL* mutations cause both DD2 and LS phenotypes; (3) codon 318 is a DD2 mutational hot spot; (4) a correlation was found between the presence of ES and the position of the mutations along OCRL domains. DD2 is distinct from LS. The mutation site and the mutation type largely determine the DD2 phenotype.

## 1. Introduction

Dent disease is a rare X-linked tubulopathy caused by mutations either in *CLCN5* (Dent disease type 1 (DD1) MIM #300009) or *OCRL* (Dent disease type 2 (DD2) MIM #300555) genes. While a role for *CLCN5* in Dent disease was suggested in 1994 [1], it was only 11 years later that *OCRL* was identified as a second disease-causing gene [2]. Pathogenic variants in the *OCRL* gene are known to cause Lowe syndrome (LS) (MIM #309000 LS) [3], while DD2 is frequently described as a mild form of LS [4]. LS is characterized by multi-organ involvement with a triad of symptoms including congenital cataracts, neurological abnormalities and a selective proximal tubular dysfunction of variable extent, while patients with DD2 mainly manifest a proximal tubulopathy [5]. Genotype–phenotype correlations conducted on a relatively small number of DD2 cases suggest that the location of DD2 mutations in *OCRL* is quite different from that of LS mutations, since most DD2 pathogenic variants were located among exons 1–7, while those responsible for the LS phenotype were found among exons 8–23 [5,6].

*OCRL* encodes for a member of the inositol polyphosphate-5-phosphatase enzyme family (OCRL), a phosphatase able to remove the 5′ phosphate group from phosphatidilinositol-4,5-bisphosphate (PIP2), a second messenger involved in vesicular trafficking [7]. Studies in Zebrafish demonstrated that a lack of OCRL1, the homolog of human OCRL, resulted in defective tubular endocytosis, a reduction in cell surface megalin expression and an accumulation of megalin in the endocytic compartments, thus supporting a role for OCRL in the recycling of this multiligand receptor [8].

The OCRL protein contains the following two major conserved domains: a central inositol polyphosphate 5-phosphatase domain and a C-terminal region with homology to the RhoGAP domain that is found in proteins that bind and stimulate the GTPase activity of Rho family small GTP-binding proteins [9]. The N-terminus of OCRL contains a PH (pleckstrin homology) domain that lacks the basic patch needed for phosphoinositide recognition and cannot directly bind to phosphoinositide-containing liposomes. Instead, binding is achieved by a loop within the PH domain containing an unconventional clathrin box linking phosphoinositide metabolism to clathrin-mediated membrane trafficking [10]. The PH domain connects to the 5-phosphatase domain by a linker region of approximately 100 amino acids, which contains an AP-2 binding site. The 5-phosphatase domain of OCRL is followed by a short helix that connects it to an ASH domain (ASPM-SPD2-Hydin) and RhoGAP. A loop in the RhoGAP domain contains a second clathrin box [11].

Dent disease is usually diagnosed by the presence of low-molecular-weight proteinuria (LMWP), hypercalciuria and often nephrocalcinosis/nephrolithiasis and chronic kidney disease (CKD) [12], but it is widely accepted that phenotypic heterogeneity characterizes the disease. Even if the phenotypic heterogeneity observed in Dent disease patients can be attributed to a proximal tubular defect [13], DD2 subjects also present with extra-renal signs such as milder cognitive, behavioral impairments, a mild increase in lactate dehydrogenase (LDH) and/or creatine kinase (CK) levels, presumably reflecting the expression of OCRL in the brain and skeletal muscle. In addition, *OCRL* mutated males may present with growth defects and bone abnormalities [14]. However, patients with DD2 lack the typical facial findings, behavioral features, metabolic acidosis, and congenital cataracts of classical LS [15].

It is still unclear whether DD2 has a clinical picture distinct from LS, and what determines the final phenotype. Due to the rarity DD2 disease and the low number of cases reported to date in comparison to DD1, the spectrum of clinical presentations in DD2 patients remains less well-defined. Here, we describe the phenotypes of 35 males clinically diagnosed with DD2 and genetically confirmed with pathogenic variants in *OCRL* in order to better define the clinical picture of the disease. Furthermore, to shed light on whether a clinical expression of a DD2 phenotype identifies a mild form of LS or instead represents a distinct phenotype due to *OCRL* gene pleiotropism, we collected and analyzed clinical and genetic data of all published DD2 patients.

## 2. Materials and Methods

### 2.1. Subjects

Index patients from twenty-five unrelated families with a clinical diagnosis of Dent disease and confirmed as DD2 by molecular diagnosis were retrospectively enrolled for this study. Ten families had two siblings affected, bringing the total of enrolled patients to 35. 

Twenty-three patients were referred for molecular diagnosis to the Laboratory of histomorphology and molecular biology of the kidney (Italy) and 12 were referred to the Rare Kidney Stone Consortium (RKSC) in the USA. Sanger sequencing was used to determine and verify the pathogenic variants in *OCRL* in our cohort [16]. *OCRL* variants were classified as pathogenic or likely pathogenic according to American College of Medical Genetics and American College of Pathologists (ACMG/AMP) guidelines [17].

### 2.2. Assessment of Phenotypic and Genetic Features

Clinical data at the time of clinical DD2 diagnosis were retrieved from Dent Disease Registry assembled by Rare Kidney Stone Consortium (*n* = 19) or from the referring clinicians from different nephrological centers in Italy. Abstracted data included anthropometrical and biochemical parameters as well as clinical data such as nephrocalcinosis, nephrolithiasis, family history of nephropathy and presence of extra-renal symptoms. Urine analyses were performed on spot samples or on 24-hour urine collections. Since units of measurement were reported for urine and serum biochemical parameters, we converted these continuous data into the following categorical variables: LMWP, albuminuria, proteinuria, hematuria, nephrotic syndrome, hypercalciuria, hypophosphatemia, hyperphosphaturia, glycosuria, acidosis and aminoaciduria. Height was compared to reference tables, and failure to thrive was defined as a value below the 50th percentile. eGFR was calculated using the “Bedside Schwartz” formula [18] and CKD was classified according to K/DOQI CKD staging system [18]. Renal tubular reabsorption of phosphate (TRP) was considered abnormal when less than 85%. Extra-renal symptoms were categorized in three groups, i.e., ocular, muscle and CNS symptoms. 

For genotype–phenotype correlations, we considered *OCRL* mutations grouped according to the expected effect on the protein, i.e., non-truncating mutations comprising missense and in-frame mutations that are unlikely to cause a complete lack of protein, truncating mutations comprising nonsense and frameshift, which are assumed to produce no protein or truncated proteins, and splice site mutations (IVS). Furthermore, we grouped mutations according to their position in OCRL domains (UniProtKB/Swiss-Prot protein accession Q01968).

### 2.3. Statistical Analysis

Fisher’s exact test was used to compare clinical data between our cohort of DD2 patients and literature data, and to highlight differences in the presence of extra-renal symptoms among different groups. A value of *p* < 0.05 was considered as significant. Data analysis was conducted using R software version 3.6.3 [19].

## 3. Results

### 3.1. Clinical Data of Our Cohort of DD2 Patients

Table S1 contains detailed clinical and genetic data of all 35 patients. The data presented were collected at the time of a clinical diagnosis of Dent disease that was subsequently confirmed as DD2 by a molecular diagnosis. Table 1 summarizes the clinical data of the current DD2 cohort (*n* = 35).

The time to clinical diagnosis of Dent disease ranged between 3 months and 15 years of age. LMWP (100%), proteinuria (97%) and albuminuria (92%) were the most common signs. Other evidence of proximal tubulopathy included hypercalciuria (95%) and aminoaciduria (44%). Nephrotic syndrome was detected in 8/35 (30%) of the patients.

Nephrocalcinosis was identified in 42% of the patients, nephrolithiasis in 32%, and hematuria in 59%. The majority of the patients had CKD at the time of the clinical diagnosis (22/23), but 50% were stage 1 and 35% were stage 2 according to the K/DOQI CKD staging system. One patient presented with stage 3B CKD at age 14. Failure to thrive was identified in as many as 94% of the patients with a severe growth retardation (below 5th percentile height) in 68% of cases.

Extra-renal symptoms were present in more than 60% of our patients (22/32). Ocular symptoms were identified in 9 out of 23 patients (39%). CNS symptoms were detected in 46% of the patients (13/28), and muscular abnormalities in 64% (14/22) (Table 1). Congenital cataracts were absent, and developmental delay was the most frequently reported sign of CNS involvement, whereas an elevated serum level of CK and/or LDH was the only reported muscular abnormality (Table S1).

Only three patients had symptoms related to muscle, CNS and eyes, one was an individual who carried both *OCRL* and *CLCN5* pathogenic variants, the other two were brothers carrying the recurrent p.(Arg318Cys) *OCRL* variant. Half of the patients that manifested extra-renal signs (11/22) had only one organ system involved.

**Figure 1:**
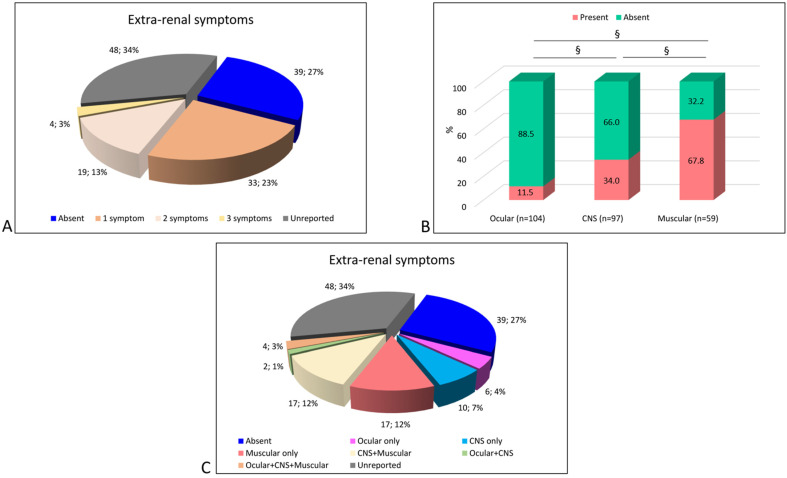
Extra-renal symptoms in DD2 patients. (**A**) Distribution by number of extra-renal symptoms presented by each subject (number of patients; percentage). (**B**) Graph bar showing the percentage of ocular, CNS or muscular involvement in DD2 patients. Fisher’s exact test § *p* < 0.01. (**C**) Detailed description of extra-renal symptoms’ combinations in DD2 patients (number of patients; percentage).

To determine the severity of the extra-renal symptoms in DD2, and to establish what extra-renal symptoms are more frequent in DD2 patients, we examined the frequency of ocular, CNS and muscular symptoms among all the published data. We reviewed all publications that reported patients clinically identified as DD2. Together with our cohort, a total of 143 DD2 subjects were included in the analysis (Table S2). In the combined cohort, we found that extra-renal symptoms were present in 39%, absent in 27%, and unreported in 34% (Figure 1A).

Ocular symptoms were rarely present in the DD2 patients (11.5%), whereas muscular symptoms were significantly more frequent than CNS symptoms (Figure 1B). The majority of the patients only had one extra renal symptom (33/143, 23%) (Figure 1A), muscular manifestations being the most common (17/33, 52%) (Figure 1C), with elevated serum CK and/or LDH the sole manifestation in most of these.

### 3.2. Histopathological Data of Our Cohort of DD2 Patients

Seven out of the 35 DD2 (20%) patients underwent a kidney biopsy on suspicion of a glomerulopathy. Almost all our patients presented with morphological abnormalities within both the glomerular (6/7) and tubular compartments (5/7), with a preponderance of global sclerosis and interstitial fibrosis (Table 2).

A few patients had proliferative lesions such as mesangial expansion or interstitial inflammation. The morphological picture of the glomeruli, mainly characterized by focal global glomerulosclerosis (FGGS), is further supported by electron microscopy data.

Glomerular lesions are also reported in 12/14 published DD2 biopsies, although the type of lesions was largely inflammatory such as mesangial proliferation. Unexpectedly, tubulointerstitial morphological lesions were less often described in these biopsies, even though DD2 is a tubulopathy. Since a biopsy is usually performed for proteinuria before a clinical suspicion of DD2 is made, more attention could have been paid to the glomerulus by the pathologist and/or person abstracting data for publications.

### 3.3. Genetic Data of Our Cohort of DD2 Patients

Table S1 details the genetic data of our 35 patients. It comprises 25 unrelated families with a total of 20 *OCRL* mutations from exon 3 to exon 19 including two novel mutations in exons 5 (c.309_310delCT, p.(His103Glnfs*27)) and 7 (c.543delC, p.(Ser183Glnfs*2)) (in bold in Table S1). Eleven out of twenty mutations are missense, exclusively localized in exons 10–12 encoding the 5-phospatase domain, while all but one mutation leading to a premature stop codon (*n* = 8) were localized in exons 3–7 encoding the PH domain. One was an in-frame deletion involving the entire exon 6. Three mutations were shared by eight unrelated families, in particular the missense p.(Arg318Cys) by four families, three coming from different geographical areas of Italy and one from the USA, suggesting the absence of a common ancestor. *OCRL* codon 318 seems to be a hot spot of missense mutations in our cohort, being involved in eight unrelated families. Forty per cent of our families had at least one affected sibling and 50% had a positive family history (Table 1).

### 3.4. Mapping DD2 Mutations in the OCRL Gene and OCRL Domains

To better define the distribution of DD2 mutations along the *OCRL* gene and OCRL protein domains, we surveyed all the *OCRL* mutations detected in the patients that were clinically defined as having DD2 disease in the literature (Table S3), including our families.

**Figure 2:**
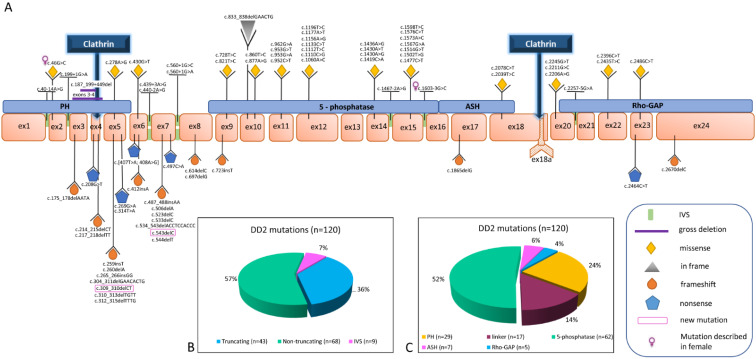
*OCRL* mutations described in DD2 subjects. (**A**). Genogram showing *OCRL* mutations in DD2 cases. Numbering is according to the cDNA sequence (GenBank entry NM_000276.4). The (**A**) of the ATG of the Methionine initiation codon is defined as nucleotide 1. (**B**) Distribution of DD2 mutations by type: truncating (nonsense and frameshift), non-truncating (missense and in-frame) or splice site mutations (IVS). (**C**) Distribution of DD2 mutations by OCRL protein domain affected.

Seventy-nine different mutations have been detected in 120 families, spanning from exon 1 to exon 24 (Figure 2A). Fifty seven percent are non-truncating and 36% are truncating mutations, the majority (52%) are localized in the 5-phosphatase domain (Figure 2B,C). Twelve are recurrent mutations, including p.(Arg318Cys) that has been reported in 13 and p.(Arg318His) that has been reported in six unrelated families.

**Figure 3:**
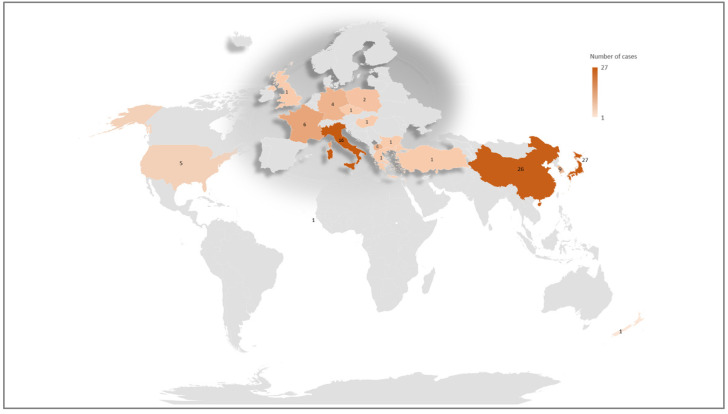
Geographical distribution of DD2 families. Number of cases for each nation are shown in the map. Of the 120 families, 12 cases were not shown in the map due to unknown geographical origin (*n* = 5) or to not-well defined nationality (*n* = 7).

To verify whether a founder effect might have accounted for so many families sharing the same mutation, we reconstructed the geographical provenance of DD2 probands. This is reported in Figure 3. As shown, the DD2 patients in the current cohort and the published literature were identified mainly in Italy, Japan and China.

**Figure 4:**
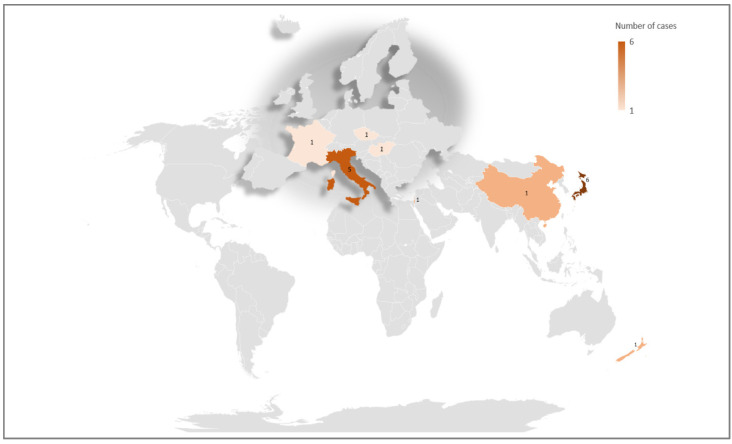
Geographical distribution of patients carrying an Arg318 mutation. Number of cases for each nation are shown in the map. Of the 20 unrelated cases, 3 were not shown in the map due to unknown geographical origin (*n* = 2) or to not-well defined nationality (*n* = 1).

Of the 20 families carrying Arg318 mutations, 17 were from six different countries, confirming that there is no founder effect but rather that codon 318 is a mutational hot spot for Dent disease type 2 (Figure 4).

Figure 2A shows the distribution of *OCRL* mutations along the gene. All the truncating mutations except three (nonsense, frameshift and gross deletions), and six splice-site mutations (IVS) cluster in the 5′ region of the gene (exons 1–9), which primarily encodes the PH domain. Missense mutations, except three, map in the 3′ region (exons 9–23), encoding the OCRL catalytic domains, thus confirming what was previously seen in smaller samples of DD2 patients, including our cohort. Interestingly, the mutations in the 5-phosphatase domain are almost exclusively missense.

Truncating mutations are expected to lead to the loss of almost all of the protein including the PH domain, 5-phosphatase and the ASH-RhoGAP module that regulates most of the known protein–protein interactions and has a key role in membrane recruitment. Eight missense and three truncating mutations map in this last module.

### 3.5. Genotype–Phenotype Correlation in Dent Disease 2

**Figure 5:**
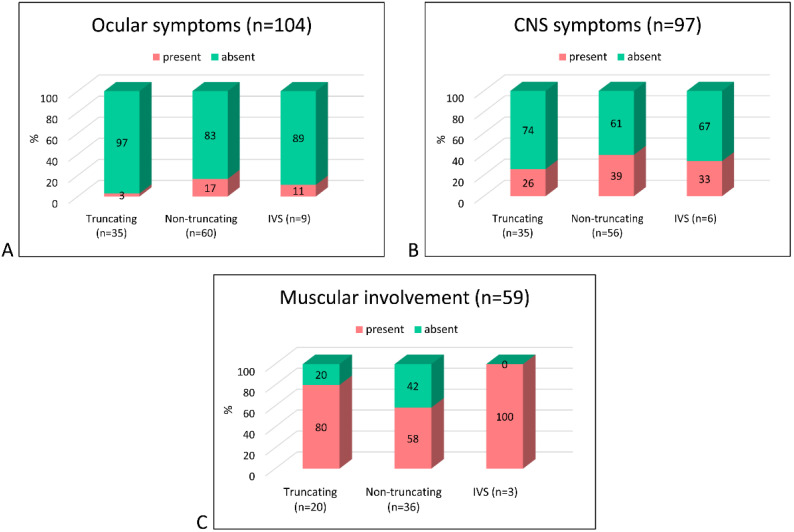
Extra-renal symptom distribution by type of *OCRL* mutation in DD2 patients. Graph bar showing the distribution of ocular (**A**), central nervous system (CNS) (**B**) and muscular (**C**) symptoms in DD2 patients.

**Figure 6:**
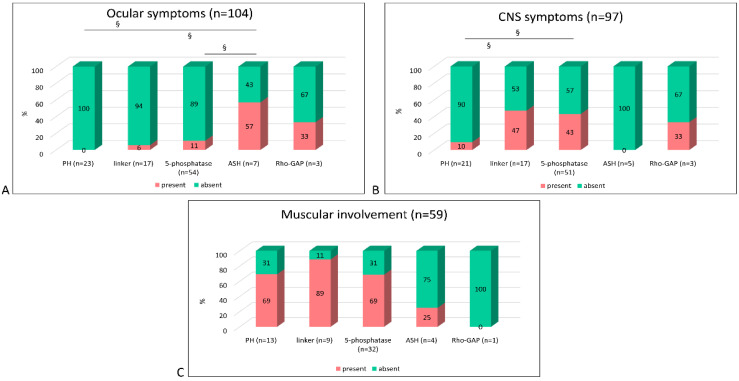
Extra-renal symptom distribution by OCRL protein domains in DD2 patients. Graph bar showing the distribution of ocular (**A**), central nervous system (CNS) (**B**) and muscular (**C**) symptoms in DD2 patients. Fisher’s exact test § *p* < 0.01.

To determine if there is a correlation between the type of mutation and the appearance of extra-renal symptoms, we analyzed the distribution of extra-renal signs according to the type of mutations (truncating vs. non-truncating vs. IVS) and their localization in the OCRL domains (Figure 5 and Figure 6).

No significant difference in the frequency of extra-renal signs was observed except for a near significance (*p* = 0.05) of ocular symptoms for non-truncating vs. truncating mutations (Figure 5). Instead, significant differences were observed among OCRL domains independently from the mutation type (Figure 6). We found that ocular symptoms were more frequently present when mutations affected the ASH domain compared to those affecting the PH and 5-phosphatase (*p* < 0.01) domains. CNS symptoms were significantly more frequent in patients carrying mutations in the 5-phosphatase and linker domains in respect to the PH (*p* < 0.01).

Muscular symptoms were not statistically analyzed due to the low number of affected cases, but, as Figure 6 shows, most patients with muscular signs carried mutations in the PH, linker or 5-phosphatase domains.

## 4. Discussion

In this study, we analyzed the genotypic and phenotypic data of the largest cohort of DD2 patients reported to date and made genotype–phenotype correlations by analyzing these and all the DD2 cases published in the literature.

Histopathological data from our cohort and those from the literature (total *n* = 21 renal biopsies) confirm that glomerulopathy is a frequent finding in DD2, as in DD1 [9]. It is known that OCRL is widely expressed in human glomeruli in podocytes, mesangial and endothelial cells, and that close interaction between OCRL and CD2AP, a protein involved in slit diaphragm maintenance in podocytes, has been demonstrated [9]. Pathogenic variants in the *OCRL* gene could disrupt this mechanism, inducing glomerular damage as a result.

As previously reported, the tubulopathy in the patients with DD1 and DD2 is similar, including aminoaciduria and metabolic acidosis typical of LS phenotype [9]. However, the age of clinical diagnosis seems to be earlier than in DD1 [9] as well as a greater frequency of familial cases, which is consistent with a greater degree of penetrance of clinical signs. Among the serious clinical aspects, failure to thrive is present in 94% of our cases, even in a severe form. Indeed, this is a typical feature of the patients with LS. A deficiency of growth hormone is not reported in DD2 as has been reported for some patients with DD1 [9], although growth defects were well documented in Ocrl KO mice [20]. CKD stage II–V was recently reported in 28% of DD2 patients [5], CKD stage II–III was also observed in 26% of the patients, despite their relatively young age.

A more severe presentation of DD2 is observed compared to DD1 due to the presence of extra-renal symptoms, which are almost absent in DD1 [9]. More than 60% of the DD2 patients in our cohort (6% without available data) had extra-renal symptoms, mostly involving a single organ system, compared to only 39% in the complete cohort (although 34% lacked data in the larger cohort analysis). Thus, extrarenal manifestations appear to affect the majority of the DD2 patients. Muscular symptoms are the most frequently reported extrarenal finding in the DD2 patients (52%), although the amount of missing data is higher than for the other extrarenal symptoms. Muscular involvement appears largely subclinical and almost exclusively manifests as elevated levels of LDH and/or CK.

The distinctive extra-renal manifestation of LS, which is described in 100% of the patients is the presence of bilateral congenital cataracts [21]. Congenital cataracts were absent in our DD2 cohort and present in only one published child [22]. In two other reported cases, cataracts were described without indicating whether it was congenital [4,23]. These two cases also presented CNS signs and, in one of them, an increase in serum LDH was also reported, suggesting that these two cases could be better classified as LS.

The overall clinical picture of DD2 patients suggests that their phenotype may be a mild form of LS [15]. Analysis of the distribution of mutations along the *OCRL* gene in small cohorts of patients [5,6] has shown that the majority of DD2 mutations mapped within the 5′ region while the LS ones mapped in the 3′ region of the gene, as if to suggest a form of gene pleiotropism, i.e., different mutations in the same gene cause different phenotypes. This hypothesis is now supported by data from the current cohort of 35 DD2 patients, and by the larger series that includes all the published cases for a total of 143 DD2 patients. Only a small number of *OCRL* mutations (*n* = 8) causes both DD2 and LS phenotypes and the hot spot codon 318 is affected only in DD2 patients. Indeed, only five mutations appear clearly shared by patients with a DD2 or LS phenotype (Supplementary Table S2). Hichri et al. [6] reported one LS patient carrying p.(Arg318Cys) who manifested CNS symptoms without ocular signs; there was no mention of muscular involvement. The c. 2257-5G>A variant is shared by two brothers, one with DD2 and the other with partial LS since he did not present ocular symptoms [24]. We hypothesize that these two boys could both have a DD2 (as opposed to LS) phenotype. We do not have information on extra-renal symptoms of the patient carrying the c.2464C>T nonsense variant [25] that is commonly reported in LS [5,6,26,27,28,29], and, therefore, we cannot exclude that this is a misdiagnosed LS patient. Furthermore, DD2 truncating mutations map almost exclusively in the PH and linker regions (exon 1–7), while missense mutations map in the 5-phosphatase domain of the gene, and only a few in the ASH-RhoGAP module (exons 9–24).

Zaniew et al. [5] found that mutations were mostly in the 3′ region, starting from exon 8 to exon 24, independent of the type of mutation in an analysis of genetic data from 81 LS patients. A more recent analysis of the distribution of approximately 200 *OCRL* gene mutations in LS patients [7] confirmed these results, demonstrating that LS truncating mutations exclusively map to exon 8, which is otherwise rarely affected by LS mutations, and that missense mutations in LS are predominantly localized in the 5-phosphatase domain. In Figure S1, we summarize the distribution of LS and DD2 mutations along the *OCRL* gene.

From these analyses, we conclude that the clustering of truncating vs. non-truncating mutations in different regions of the gene is peculiar to DD2. These data are challenging and seem to suggest that OCRL protein loss due to early stop codons is associated with a less severe clinical phenotype than missense mutations that presumably lead to the protein being synthetized but lacking its catalytic activity.

To test this hypothesis, we analyzed the distribution of extra-renal symptoms in DD2 patients according to *OCRL* mutation types and OCRL domains. The type of mutations does not influence the frequency of extra-renal symptoms; in particular, truncating mutations appear not to increase their frequency, further confirming the less severe impact of these mutations on the clinical phenotype. One potential explanation is the possibility that premature termination codons (PTCs) may not be fully inactivating because of the presence of a later start codon. PTCs may also be bypassed when translational readthrough allows the decoding of stop codons as sense codons, thus enabling protein translation [30], or can prompt exon skipping by altering exonic splicing enhancer (ESE) and silencer (ESS) motifs [31]. Further studies by computational analysis of the mutations should be performed to see if PTCs are predicted to affect splicing or if the mRNA context is appropriate for the translational readthrough to take place. Associated functional studies will help us to fully understand the genotype–phenotype correlation in DD2. Another explanation may lie with INPP5B (also known as Type II 5-phosphatase), an OCRL homologue with ~45% sequence identity and close structural similarity, which shares most interacting partners with OCRL. A significant level of INPP5B expression in relevant tissues may attenuate the severity of the OCRL loss-of-function phenotype [11]. In an Ocrl KO model, Jänne et al. demonstrated the overlapping functions of the two enzymes, and the compensatory role of Inpp5b [32].

Significant differences, instead, have been found between the presence of extra-renal symptoms and the position of the mutations along the different OCRL domains. Ocular symptoms are almost absent in the 143 DD2 cases. When present, they are more frequent in DD2 patients with mutations in the 3′ region of the gene—ASH and Rho-GAP domains containing the majority of LS truncating mutations (Figure S1)—suggesting a reason why ocular symptoms are more typical of LS. Elevated levels of CK and/or LDH are more frequently observed in patients with mutations in the 5′ region of the gene, the site of many DD2 mutations, suggesting that they are characteristic of DD2, while CNS symptoms more frequently manifest in patients carrying mutations in the catalytic domain of the OCRL protein where both DD2 and LS missense mutations are localized. However, in this domain, a few mutations (5 out of 8) have been associated with both diseases.

Our results suggest that the *OCRL* gene is pleiotropic. In fact, the available data suggest that DD2 and LS are two distinctive diseases largely due to different types of mutations and their position along the gene. However, when mutations affect the 3′ of the gene, DD2 should be considered a mild form of LS because of the presence of extra-renal symptoms. Nevertheless, a few mutations are shared by individuals with DD2 and LS phenotypes in the 5-phosphatase domain, and DD2 mutations rarely involve the ASH and Rho-GAP region. An explanation of these findings may come from a structural analysis of *OCRL* missense mutations conducted by Piruccello and De Camilli in 2012 [11]. They demonstrated that most LS mutations in the 5- phosphatase domain cluster in the hydrophobic core of the protein, suggesting that these mutations would be destabilizing, affecting the folding core of the protein. DD2 mutations, instead, primarily localize to surface residues at or near the catalytic site, without affecting the core of the protein.

## 5. Conclusions

Certainly, our work suffers from some limitations due to the retrospective nature of the study. These are mainly reflected in the comparison of DD2 clinical signs between our cohort and that collected from the literature because of the different methods of defining clinical signs. Nevertheless, from our results, it appears that DD2 has a distinct phenotype and genotype from LS, and that the mutation site and the mutation type largely determine the DD2 phenotype. The absence of extra-renal symptoms in DD2 is mainly associated with truncating mutations in the PH and linker domain, whereas, as in LS, ocular and CNS symptoms are mostly associated with missense mutations localized in the 5-phosphatase domain.

## Figures and Tables

**Table 1:** Clinical signs of the 35 Dent disease type 2 (DD2) subjects enrolled. Continuous variables were reported as median [Min, Max], categorical variables as number of cases (%). LMWP: Low-molecular-weight proteinuria, eGFR: estimated glomerular filtration rate, CKD: Chronic kidney disease, CNS: central nervous system, TRP: renal tubular reabsorption of phosphate, Y: present, N: absent.

Clinical Sign		DD2 (*n* = 35)
LMWP	Y	27 (100.0)
	N	0 (0.0)
Albuminuria	Y	12 (92.3)
	N	1 (7.7)
Proteinuria	Y	29 (96.7)
	N	1 (3.3)
Nephrotic syndrome	Y	8 (29.6)
	N	19 (70.4)
eGFR		90.00 [44.00, 143.00]
CKD	0	1 (4.3)
	1	13 (56.5)
	2	8 (34.8)
	3	1 (4.3)
Glucosuria	Y	8 (30.8)
	N	18 (69.2)
Aminoaciduria	Y	7 (43.8)
	N	9 (56.2)
Hematuria	Y	10 (58.8)
	N	7 (41.2)
Hypercalciuria	Y	20 (95.2)
	N	1 (4.8)
TRP	Normal	7 (63.6)
	<85%	4 (36.4)
Hypophosphatemia	Y	5 (22.7)
	N	17 (77.3)
Nephrocalcinosis	Y	13 (41.9)
	N	18 (58.1)
Urolithiasis	Y	10 (29.4)
	N	24 (70.6)
Rickets	Y	3 (10.3)
	N	26 (89.7)
Hypertension	Y	1 (3.8)
	N	25 (96.2)
Family history	Y	11 (50.0)
	N	11 (50.0)
Extrarenal symptoms	Y	22 (68.8)
	N	10 (31.2)
Ocular symptoms	Y	9 (39.1)
	N	14 (12.2)
CNS symptoms	Y	13 (46.4)
	N	15 (53.6)
Muscular abnomalities	Y	14 (63.6)
	N	8 (36.4)
Growth	Normal	2 (7.1)
	Below 50 percent	2 (7.1)
	Below 25 percent	4 (14.3)
	Below 10 percent	2 (7.1)
	Below 5 percent	18 (64.3)

**Table 2:** Histopathological data from 21 DD2 kidney biopsies. Data are shown as number of cases with each characteristic within all described cases. FGGS: Focal global glomerulosclerosis, FSGS: Focal segmental glomerulosclerosis, GBM: Glomerular basement membrane, TEM: Transmission electron microscopy.

	DD2 Cohort (*n* = 7)	Literature (*n* = 14)
Age at Biopsy	4–11 yr 11 mo	3–14 yr
Glomerular histology		
Number of glomeruli	8–75	
Normal	1/7	2/14
Unspecified sclerosis		
FGGS	4/7	1/14
FSGS	0/7	3/14
Mesangial proliferation	0/7	6/14
Minor glomerular abnormalities	0/7	1/14
Periglomerular fibrosis	0/7	
Expansion of mesangial matrix	2/7	1/14
Immature glomeruli	1/7	
Adherence to Bowman capsule	0/7	1/14
Other (Perihyliar hyalinosis, ECM hyperplasia, Collapsed tuft, Podocytes’ hypertrophy, glom lesion)	2/7	2/14
Tubular histology		
Normal	2/7	2/6
Tubular atrophy	4/7	1/6
Interstitial fibrosis	4/7	1/6
Calcification	0/7	
Tubulointerstitial lesions	0/7	2/6
Calcium deposits	0/7	
Intratubular proteinaceous casts	0/7	1/6
Interstitial inflammation	1/7	
Nephrocalcinosis	0/7	
Vascular degeneration	1/7	
Interstitial mononuclear cells infiltrate	0/7	
Interstitial lymphocytes infiltrate	0/7	1/6
Acute tubular necrosis	0/7	1/6
Other (Cortical fibrosis, Interstitial chronic inflammation, chronic tubulointerstitial nephropathy with ischemic renal damage)	1/7	
Immunofluorescence		
Negative	3/7	2/2
IgM deposits	1/7	
C3 deposits	1/7	
OTHER	3/7	
TEM		
Normal	0/6	
Foot process effacement	5/6	2/2
Electrondense deposits	1/6	
Mesangial proliferation	2/6	
Global sclerosis	2/6	
Irregular GBM folding	2/6	1/2
Other (Expansion of mesangial matrix, Microvillarization of podocytes)	4/6	

## Data Availability

All clinical and genetic data included in this study are provided in Tables S1 and S2.

## Supplementary Materials

The following are available online at https://www.mdpi.com/article/10.3390/genes12101597/s1, Table S1. Clinical and genetic data of our cohort of DD2 patients (*n* = 35), Table S2. Genetic data and extrarenal signs of DD2 patients (*n* = 143), Table S3. List of OCRL pathogenic variants reported in DD2 patients, Figure S1. Genogram showing the distribution of LS and DD2 mutations along the *OCRL* gene. Upper panel shows LS mutations described in [7], lower panel shows DD2 mutations from Table S3, References related to Table S3.
