# Supplementary material for: Genotype Phenotype Correlation in Dent Disease 2 and Review of the Literature: OCRL Gene Pleiotropism or Extreme Phenotypic Variability of Lowe Syndrome?
Source: Genes (Basel). 2021 Oct 11;12(10):1597. doi: 10.3390/genes12101597 (PMC8535715; doi:10.3390/genes12101597)
Supplement: Supplementary file 1 [file genes-12-01597-s001.zip › genes-1384973-supplementary.pdf]

Supplementary Material

Table S1. Clinical and genetic data of our cohort of DD2 patients (n=35). Patients carrying new mutations are highlighted in bold. Y: present, N: absent, N/A: data not available, β2MG: urinary beta-2 microglobulin, RBP: retinol binding protein, CNS: central nervous system, CK: creatin kinase, LDH: lactate dehidrogenase. eGFR was estimated using the Bedside Schwartz formula; ESRD end stage renal disease; PTH Parathyroid hormone; BUN blood urea nitrogen

#	DOB	Genotype	AA change	Age at diagnosis	Age of molecular diagnosis	weight	height	Growth height
34	11/10/2004	large deletion/complex rearrangement Ex3 c.187_199+449del	p.Arg63fs	11yrs	11yrs 2mo	34,5	123	Below 5
33	05/08/2011	Ex 5 c.260_260delA	p.Gln87Argfs*19	2yrs	3yrs 9mo	N/A	N/A	N/A
**1**	**1961**	**Ex5 c.309_310delCT**	**p.His103Glnfs*27**	**14**	**48**	**39**	**145**	**Below 5**
2	1990	Ex5 c.314T>A	p.Leu105*	13		31,9	137	Below 5
23	16/07/2002	IVS6 c.439+3A>G r.350_439 del Ex6 inframe	p.Arg100_Gly129 del	6y 4mo		20	108	Below 5
3	1999	Ex7 c.533delC	p.Pro178Hisfs*6	7y 1mo		30	118	Below 25
4	2009	Ex7 c.533delC	p.Pro178Hisfs*6	1 y 7 m		10,95	79	Below 25
5	**2003**	**Ex7 c.543delC**	**p.Ser183Glnfs*2**	**13**		**39**	**137**	**Below 5**
36	14/05/2012	IVS7 c.560+1G>C	p.Met187fs	6yrs	6yrs	14,7	98	Below 5
6	1994	Ex10 c.860T>C	p.Phe287Ser	10		30	125	Below 5
8	2009	Ex11 c.952C>T	p.Arg318Cys	5y 5mo			Normal	N/A
9	04/04/1992	Ex 11 c.952 C>T	p.Arg318Cys	17 y 9 m	17 y 9 m	61	165,5	Below 10
10	04/08/1996	Ex 11 c.952 C>T	p.Arg318Cys	13 y 5 m	13 y 5 m	65	149	Below 10
7	1995	Ex11 c.952C>T	p.Arg318Cys	10y 3mo		38	135,8	Below 50
28	31/03/1999	Ex11 c.952C>T	p.Arg318Cys	15yrs 8m	16yrs 6mo	40,4	141,4	Below 5
29	09/10/2001	Ex11 c.952C>T	p.Arg318Cys	14yrs 6 mo	14yrs 6mo	40,6	144	Below 5

11	2002	Ex11 c.953G>A	p.Arg318His	9		28,8	121,5	Below 5
12	2007	Ex11 c.953G>A	p.Arg318His	5		19,5	107	Below 50
13	16/03/2009	Ex 11 c.953G>T	p.Arg318Leu	5	5	17	97,9	Below 5
14	21/03/2005	Ex 12 c.1133C>T	p.Ala378Val	6	9	23,2	122,5	Below 5
15	09/08/2007	Ex 12 c.1133C>T	p.Ala378Val	4	7	19,5	111	Below 5
26	23/02/2007	Ex12 c.1156A>G	p.Arg386Gly	7yrs 5 mo (sympotatic age 4)	8yrs 8m	16,4	102,6	Below 5
27	19/09/2000	Ex12 c.1156A>G	p.Arg386Gly	13yrs 6mo	15yrs	38,7	149,7	Below 5
16	18/09/2002	Ex15 c.1477C>T	p.Arg493Trp	9y 3m	9y 6m	31,5	129	Below 25
17	2001	Ex15 c.1567G>A	p.Asp523Asn	4y7mo		16	104	Below 50
18	2006	Ex15 c.1567G>A	p.Asp523Asn	1 y 9 m		9,6	78,5	Below 5
19	04/09/1994	Ex15 c.1567G>A	p.Asp523Asn	14 y 10 m	15 y 6 m	92.7 (@17y10mo)	162.6 (@17y10mo)	Below 5
20	16/05/2000	Ex15 c.1567G>A	p.Asp523Asn	2y	9y 4m	68 (@14y7mo)	153 (@14y7mo)	Below 5
30	14/09/2000	E15 c.1573A>C	p.Lys525Gln	14yrs 4mo	14yr 5mo	64,2	152,2	Below 25
31	09/04/2002	E15 c.1573A>C	p.Lys525Gln	13yrs 2mo	13yr 8mo	48,8	145,7	Below 5
35	14/05/2016	Ex17 c.1865delG	p.Gly622Afs*22	3mo	1yr 6mo	10	74	Below 5
24	05/09/2004	Ex18 c.2078C>T	p.Pro693Leu	8 yrs	8yrs	46,6	128	Normal
25	09/08/2005	Ex18 c.2078C>T	p.Pro693Leu	7yrs 2 mo	7yrs 5 mo	23	114	Below 5
21	N/A	Ex19 c.2211G>C	p.Glu437Asp	10		27,5	118,8	Below 5
22	N/A	Ex19 c.2211G>C	p.Glu437Asp	11		27,5	125	Below 5

Table S1. (Continue)

#	LMWP	albuminuria	proteinuria	Nephrotic Syndrome	calciuria	hypercalciuria	serum creatinine	eGFR	CKD	ESRD
34	β2MG 780 mg/L; 230769 ug/g	288 mg/L (in 2013, 9yrs)	2024mg/24h	N	7.1 24h	N/A	0.58mg/dL	88	2	N/A
33	β2MG333mg/L, α2MG:539 mg/L	1900 mg/L	1,26	N	10.1 mg/dL	Y	0.5 mg/dL	N/A		N/A
**1**			**Y**		**N/A**		**N/A**	**N/A**		**N**
2	β2MG 12500ug/24h		1300mg/24h	N	N/A			N/A		
23	β2MG 94750ug/L	1 g/24h			7.7 mg/kg/24h	Y	0,56 mg/dL	80	2	N
3	β2MG 15300ug/24h		623mg/24h	N	5.8 mg/kg/24h	Y		N/A		
4	N/A	N/A	150mg/dl	N	N/A		0,26 mg/dL	125	1	N
**5**	**Y**		**62 mg/kg/24h**	**N**			**55 umol/L (0,63 mg/dL)**	**90**	**1**	
36	N/A	N/A	N/A		N/A		N/A	N/A		N/A
6	β2MG 81567ug/24h	70.3 mg/24h	690mg/24h	N	4.1 mg/kg/24h	Y	0,64 mg/dL	81	2	N
8	Y	100-300 mg/dl	1.4-4.4 mg/mg	?	N			N/A		
9	β2MG >4mg/L		1.47 mg/mg - 1385 mg/m^2/24h	Y	0,30 mg/mg	Y	0,75 mg/dL	91	1	N
10	β2MG >4mg/L	1,01 mg/mg - 634,63mg/mq/24h	2.06 mg/mg - 1292.7 mg/mq/24h	Y	0.38 mg/mg	Y	0,9 mg/dL	68	2	N
7	β2MG 85600ug/24h		1200 mg/24h	N	3-7 mg/kg (iper)	Y		N/A		N
28	RBP: 3455 mc/L; β2MG100mg/L	117.1 mg/L	147mg/dl	N	0.8 mmol/L (3.21 mg/dl)	Y	82 umol/L (0.93mg/dl)	63	2	N
29	N/A	N/A	1.92, 2.03g/24 h	N	2.28mmol/L (9.14 mg/dl)	Y	58umol/L (0.66 mg/dl)	90	1	N
11	tubular proteinuria	N/A	Y		Y		0,36 mg/dL	143	1	N
12	β2MG 50000 ug/24h	90.2 mg/24h	560 mg/24h	N	6.35 mg/kg/24h	Y	0,43 mg/dL	103	1	N
13	β2MG 123000 ug/L	266,7 mg/g	5.5 mg/mg	Y	0.9 mg/mg	Y	0,45 mg/dL	90	1	N

14	110000 ng/ml		2.3 mg/mg-978mg/mq/24h	Y	0,26 mg/mg	Y	0,39 mg/dL	130	1	
15	100000 ng/ml		2.2 mg/mg-473 mg/mq/24h	N	0,12 mg/mg	N	0,32 mg/dL	143	1	
26	RBP: 111000 mcg/L 2013	N/A	137.8 mg/dL in medical records 2011 3yrs	N	51 mg/dl	Y	0.37 md/dL @2011 3yrs (0.39 at 2013 6yrs)	115,0	1,0	N
27	RBP: 147000 mcg/L	N/A	N/A		29.2 mg/dl	Y	0.71 mg/dL	87	2	N
16	β2MG >4mg/L	0.38 mg/mg - 228,54 mg/mq/24h	1140 mg/mq/24h	Y	6.8 mg/kg/24h	Y	0,42 mg/dL	127	1	N
17	β2MG 53900ug/24h		960mg/24h	N	N/A			N/A		
18	N/A	13 mg/dl	459mg/24h	N	N/A		0,26 mg/dL	125	1	N
19	β2MG >4mg/L		1.66 mg/mg - 1637 mg/mq/24h	Y	0.45 mg/mg	Y	1,53 mg/dL	44	3	N
20	β2MG >4mg/L		2.15 mg/mg	Y	0.38 mg/mg	Y	0,88 mg/dL	72	2	N
30	β2MG 0.52 mg/L	N/A	0.99 mg/dl	N	3 mg/dL	Y	0.5 mg/dL	126	1	N
31	N/A	N/A			12 mg/dL	Y	0.5 mg/dL	120	0	N
35	N/A	N/A	N/A		N/A		N/A	N/A		N/A
24	β2MG 127333 mcg/L	45000 mg/L	Y; 194 mg/dL	N	10mg/dl			N/A		N
25	β2MG 176812 mg/L	14.1 mg/L	448 mg/dL	Y	35.7 mg/dL (two weeks later 8.1mg/dl)	Y	0.6 mg/dL	78	2	N
21	β2MG 14500ug/24h		2100mg/24h	N	N/A	Y		N/A		
22	β2MG 16300ug/24h		1530mg/24h	N	N/A	Y		N/A		

Table S1. (Continue)

#	serum albumin	serum potassium	PTH	acidosis/ HCO_3	serum phosphate	Hypophosp hatemia	Hyperphos phaturia	TRP or TmP/GFR	glycosuria	aminoaciduria	BUN	Hematological abnormalities
34	4.5 g/dL	4.1 mmol/L	N/A	22mmol/ml	4.7 mg/dL	N	N/A	N/A	N/A	N/A		N/A
33	4.5 gr/dl	4.6mEq/L	N/A	N/A	5.8mg/dL	N	N/A	N/A	N/A	N/A		N/A
**1**	**N/A**	**N/A**	**N/A**	**N/A**	**N/A**	**N/A**	**N/A**	**N/A**	**Y**			
2			18 ng/l	26 mmol/l	1.26 mmol/l	N			Y	Y		
23			N	N		Y	N	85%	N	Y		
3			3 pg/ml	26 mmol/l	1.45 mmol/l	N			Y	Y		
4	3,95 g/dL	4.12 mEq/L	14 pg/ml	24.3mEq/L	5.38 mg/dl	N/A		N/A	N	N/A		N
**5**	**44 g/L**	**3.9mmol/L**			**1.48 mmol/L**		**Y**					
36	N/A	N/A	N/A	N/A	2.5 mg/dL	Y	N/A	N/A	N/A	N/A		N/A
6		4	36 ng/l	26 mmol/l	2.5 mmol/l	N		N/A	N	N		N
8								>82%	N	N		
9	50,5 g/dL	4,9	64,2 pg/ml	23	4 mg/dl			83%	N	N		N
10	56 g/dL	4,4	80,3 pg/ml	22,6	4.6 mg/dl			80%	N	N		N
7			26 pg/ml	25	4 mg/dl	N		78%	N	ND		
28	40g/L	4.4mmol/L	N/A	N/A	3.99mg/dL (1.29 mmol/L)	N	N/A	N/A	N/A	N/A		N
29	33g/L	3.6 mmol/L	N/A	N/A	1.30 mmol/L	N	N/A	N/A	N/A	N/A		N
11		5,3	38 ng/l		N/A	N		N/A	N	N		N
12		3,8	N/A		1.53 mmol/l	N		N/A	N	N		N
13	51 g/dL	3,6	25 pg/ml	23,2	4,53 mg/dl			83,6%	N			N
14	42 g/dL	4,0	26 pg/ml	23,6	5,4 mg/dl				N			

15	43,1 g/dL	3,9		24,1	5,1 mg/dl				N			
26	4.5 @10/2011 (4yrs)	4.1mmol/L @10/2011 (4yrs)	N/A	20mmol/L	N/A	N/A	N/A	N/A	N	N/A	16mg/dL	N
27	N/A	N/A	N/A	N/A	4.6 mg/dL	N	N/A	N/A	N/A	N/A	N/A	N
16	49	4,6	51 pg/ml	25 mEq/L	5.2 mg/dl	N		85%	N	N		N
17			N/A	25 mmol/l	1.58 mmol/l	N			N	Y		
18	4,08	3.7 mEq/L	N/A	22.5mEq/L	5.11 mg/dl	N/A		N/A	N	N		N
19	49,2	4,1	4,1 pg/ml	24,8	3.5 mg/dl			49%	Y	Y		N
20	42	4,1	15,9 pg/ml	22,5	5.7 mg/dl			85%	Y	Y		N
30		4.8 in 2016 mEq/L		28mmol/L in 2016	4.6 mg/dL	N	N/A	N/A	N	N/A		N
31	4.4mg/dl	N/A	15 pg/ml	28mmol/L	4.4 mg/dL	N	N/A	N/A	N/A	N/A		N
35	N/A	N/A	N/A	N/A	1.19 mmol/L	Y	N/A	N/A	N/A	N/A		N/A
24	4.5 g/dL	3.9 mEq/L		20 mEq/L	4.6mg/dL	N	N/A	N/A	N	N/A	38 mg/dl	N/A
25	4.6 g/dL	3.5 mEq/L		24 mEq/L		N		N/A	Y		25mg/dl on 11/2013	N/A
21			20 ng/l	Acidosis 17 mmol/l	1.13mmol/l	Y		79%	Y/N	N		
22			25 ng/l	Acidosis 17 mmol/l	1.13 mmol/l	Y		89%	Y/N	Y/N		

Table S1. (Continue)

#	rickets	Hema turia	Nephrocalc inosis	Nephro/ Urolithiasis	Family History	Hypertension	cataract	ocular abnormalities	CNS	CK (units)	LDH (units)
34	N	N	N	N	Y father and father's brother kidney stones	N	N/A	N/A	N	N/A	N/A
33	N	N	N/A	N	N	N	N/A	N/A	N	N/A	319 U/L
**1**	**N**	**Y**	**N**	**N**		**N**	**N**	**N**	**developmental delay**	**N**	
2	N		N	N	Y		N	N	N	368 U/L	
23	Y		N	N	Y	88/44		Y (glaucoma)	attention deficit hyperactivity disorder (ADHD), mild mental retardation	Y	Y
3	N		N	N	Y		N	N	Y	336 U/L	
4	N	N	Y	N	Y	N	N	N	Hypotonia, psycho-motor and language delay	302 U/L	468 U/L
5		**Y**	**N**	**N**		**N**			**developmental delay, hypoacusia**	**Y**	
36	N/A	Y	Y	Y	N	N/A	N/A	N/A	N/A	N/A	N/A
6	N	Y	Y	N	Y	N	N	N	learning and behavioural impairments	N	456 U/L
8			N				N	N	N		
9	N		N/A	N		N	N	Y (corneal abrasion)	developmental delay	159 U/l	447 U/l
10	N		N	N		N	N	Y (thickening of the lens)	developmental delay	397 U/L	685 U/L
7	N		Y	N	N					180 UI/L	
28	N	N/A	N/A	N	N	N	N/A		Y "squint" delay onset at 5yrs	N/A	N/A
29	N	N/A	N	N	N	N	N/A	N/A	N	N/A	N/A

11	N	Y	Y	Y		Y	N	N		Y	N/A
12	N	Y	Y	N		N	N	Y (retinopathy type 1)			N/A
13	N		Y (4 yrs)	Y (4 yrs)		N	N	N	N	293	369
14	N		Y	Y		N	N	N	N	151 U.I./L	271 U/L
15	N		N	N		N	N	N	N	110 UI/L	289/L
26	N	Y	N	Y (@ 2014)	N	N	N/A	N/A	N	N/A	N/A
27	N	N	N/A	Y	N	N	N/A	N/A	N	N/A	N/A
16	N	Y	Y	N		N	N	N	developmental delay, hyperactivity	274 U/L	630 U/L
17	N		N	N	N			Y (glaucoma)	N	238 U/L	
18	N	N	N	N	Y	N	N	N	N	444 U/L	421 U/L
19	Y		Y 3 y 9 m	y 17 y 4 m		N	N	N	developmental delay	684 U/L	655 U/L
20	Y		8y	y 10y 4m		N	N	N	developmental delay	307 U/L	627 U/L
30	N	N	N	N	Y	N on an ACEi	N/A	N/A	N	N/A	N/A
31	N	N	N	Y	Y	N on an ACEi	N/A	N/A	Attention deficit disorder (ADD)	N/A	N/A
35	N/A	N/A	N/A	Y	N	N/A	N/A	Y (glaucoma)	N/A	N/A	N/A
24	N/A	Y	N	N	N	N	N/A	N/A	N/A	N/A	N/A
25	N/A	Y	N	N	N	N	N/A	N/A	N/A	N/A	N/A
21	N		N	N	Y		N	Y (bilateral cortical opacity of the lens, retinal dystrophy)	N	N	
22	N		Y	Y	Y		N	Y (accentuation of the "Y" in the anterior segment of the eye with normal fundus)	N	N	

Table S2. Genetic data and extrarenal signs of DD2 patients (n=143). DD2 mutations and references refer to Table S3. Y: present, N: absent, N/A: not described. When present, parentheses indicate the number of patients described.

Exon/ Intron	Type of Mutation	Nucleotide	Cases (num)	Families (num)	Ocular			CNS			Muscular			Nationality	Mutation reported in LS
					Y	N	N/A	Y	N	N/A	Y	N	N/A		
1	IVS	c.40-14A>G	1	1		N			N (no hypotony)				N/A	French	
2	Missense	c.46G>C	1	1		N			N			N (both CK and LDH)		Japanese	
3	Frameshift	c.175_178delAATA	1	1		N				N/A			N/A	Japanese	
3	Gross deletion	c.187_199+449del	1	1			N/A		N				N/A		
3	IVS	c.199+1G>T	1	1		N				N/A			N/A	Japanese	
3	IVS	c.199+1G>A	2	2		N			N (1pt)	N/A (1pt)	Y (high LDH 1pt)		N/A (1pt)	Japanese, European	
3-4	Gross deletion	exons 3-4	2	2		N (1 pt)	N/A (1pt)		N				N/A	French, Chinese	
4	Frameshift	c.214_215delCT	1	1			N/A			N/A			N/A	Polish	
4	Frameshift	c.217_218delTT	2	2		N			N			N (1pt)	N/A (1pt)	European, Unknown	
4	Nonsense	c.208G>T	2	1		N				N/A			N/A	Japanese	
5	Frameshift	c.259insT	1	1		N			N				N/A	Korean	
5	Frameshift	c.260delA	5	5		N (2pts)	N/A (3pts)		N (3pts)	N/A (2pts)	Y (2pts high CK and LDH)	N (1pt)	N/A (2pts)	Korean, Turkish, Chinese	
5	Frameshift	c.265_266insGG	1	1		N			N		Y (high LDH)			Japanese	
5	Missense	c.278A>G	1	1		N			N				N/A	Japanese	
5	Frameshift	c.304_311delGAACACTG	1	1		N			N		Y (high CK)			Japanese	
5	Frameshift	c.309_310delCT	1	1		N		Y (developmental delay)				N (CK)		Italian	

Exon/ Intron	Type of Mutation	Nucleotide	Cases (num)	Families (num)	Ocular			CNS			Muscular			Nationality	Reported in LS
					Y	N	N/A	Y	N	N/A	Y	N	N/A		
5	Frameshift	c.310_313delTGTT	3	3		N (3pts)		Y (mild mental retard 1pt)	N (2pts)		Y (high CK and LDH 3pts)			Macedonian	
5	Frameshift	c.312_315delTTTG	1	1		N			N				N/A	Chinese	
5	Nonsense	c.269G>A	1	1			N/A			N/A			N/A	Chinese	
5	Nonsense	c.314T>A	1	1		N			N		Y (high CK)			Italian	
6	Frameshift	c.412insA	2	1		N			N		Y (2pts high CK and LDH)			German	
6	Nonsense	c.[407T>A; 408A>G]	1	1		N			N			N (both CK and LDH)		Japanese	
6	Missense	c.430G>T	1	1			N/A			N/A			N/A	Chinese	
6	IVS	c.439+3A>G	1	1	Y (Peripapillary optic nerve atrophy with gray papillae, glaucoma)			Y (Mild mental retardation, ADHD)			Y (high CK and LDH)			Italian	
6	IVS	c.440-2A>G	1	1		N				N/A			N/A	American	
7	IVS	c.560+1G>C	1	1			N/A			N/A			N/A		
7	Frameshift	c.487_488insAA	1	1		N			N				N/A	German	
7	Frameshift	c.506delA	3	3		N		Y (1pt mild developmental delay, 1pt mild degree of developmental delay)	N (1pt)		Y (1pt eleveted muscle enzymes in serum, 1pt muscle hypoplasia - primary miopathy)		N/A (1pt)	Korean	Y [1]
7	Frameshift	c.523delC	2	1		N			N				N/A	Chinese	

Exon/ Intron	Type of Mutation	Nucleotide	Cases (num)	Families (num)	Ocular			CNS			Muscular			Nationality	Reported in LS
					Y	N	N/A	Y	N	N/A	Y	N	N/A		
7	Frameshift	c.533delC	2	1		N		Y (DD99BS mental delay, DGL09BS hypotonia, psycomotor and language delay)			Y (high LDH x1, high CKx1)			Italian	
7	Frameshift	c.534_543delACCTCCACCC	1	1		N		Y (mild mental imparement, mild hypotonia)					N/A	Unknown	
7	Frameshift	c.543delC	1	1			N/A	Y (developmental delay, hypoacusia)			Y (high CK)			Italian	
7	Frameshift	c.544delT	1	1		N			N				N/A	Chinese	
7	Nonsense	c.497C>A	1	1		N			N				N/A	Unknown	
7	IVS	c.560+1G>A	1	1		N		Y (mental imparement)					N/A	English	
8	Frameshift	c.614delC	1	1			N/A			N/A			N/A	Chinese	
8	Frameshift	c.697delG	1	1			N/A			N/A			N/A	Chinese	
9	Frameshift	c.723insT	1	1			N/A			N/A			N/A	Chinese	
9	Missense	c.728T>C	3	2		N		Y (1pt mild developmental delay)	N (2pts)		Y (1pt muscle hypoplasia of both upper extremities)		N/A (2pts)	Korean	
9	Missense	c.821T>C	5	5		N (3pts)	N/A (2pts)	Y (1pt developmental delay)	N (1pt)	N/A (3pts)	Y (1pt high CK and LDH)		N/A (4pts)	French, Japanese, German, Chinese	Y [2]
10	In-frame	c.833_838delGAACTG	2	1		N			N				N/A	Chinese	

Exon/ Intron	Type of Mutation	Nucleotide	Cases (num)	Families (num)	Ocular			CNS			Muscular			Nationality	Reported in LS
					Y	N	N/A	Y	N	N/A	Y	N	N/A		
10	Missense	c.860T>C	1	1		N		Y (neurological and behavioural impairment)			Y (high LDH)			Italian	
10	Missense	c.877A>G	1	1		N			N			N (both CK and LDH)		Japanese	
11	Missense	c.952C>T	17	13	Y (1pt thickening of the lens, 1pt corneal abrasion)	N (11pts)	N/A (4pts)	Y (2pts mild mental retardation; 2pts developmental delay, 1pt this paper: "squint" delay, 1pt normal except communication skills, 1pt ubelical hernia)	N (7pts)	N/A (3pts)	Y (1pt high LDH, 2pts high LDH, 2pts high CK and LDH, 1pt high CK, 1pt mild motor development delay	N (3pts)	N/A (7pts)	French, Japanese, European, Italian, Czech, Hungarian, Israelian	Y [2]
11	Missense	c.953G>A	6	6	Y (1pt retinopathy type 1)	N (4pts)	N/A (1pt)		N (2pts)	N/A (4pts)	Y (1pt)	N (1pt both CK and LDH)	N/A (4pts)	Japanese, Chinese, Unknown, Italian	
11	Missense	c.953G>T	1	1		N			N		Y (high LDH)			Italian	
11	Missense	c.962G>A	1	1	Y (clinically not apparent cataract with no visual impairment)			Y (mild mental imparement)					N/A	Unknown	Y [3]
12	Missense	c.1060A>C	1	1		N			N				N/A	French	
12	Missense	c.1110C>G	1	1			N/A			N/A			N/A	Chinese	
12	Missense	c.1112T>C	1	1			N/A			N/A			N/A	Chinese	
12	Missense	c.1133C>T	2	1		N			N			N		Italian	
12	Missense	c.1156A>G	2	1			N/A		N				N/A	Unknown	

Exon/ Intron	Type of Mutation	Nucleotide	Cases (num)	Families (num)	Ocular			CNS			Muscular			Nationality	Reported in LS
					Y	N	N/A	Y	N	N/A	Y	N	N/A		
12	Missense	c.1177A>T	1	1	Y (cataract, early ocular density)			Y (not formally tested clinically impaired cognitive function, depression)			Y (high LDH)			European	
12	Missense	c.1196T>C	1	1			N/A			N/A			N/A	Chinese	
14	Missense	c.1419C>A	1	1			N/A			N/A			N/A	Chinese	
14	Missense	c.1430A>G	1	1			N/A			N/A			N/A	Chinese	
14	Missense	c.1430A>T	3	1		N				N/A			N/A	Japanese	
14	Missense	c.1436A>G	1	1		N		Y (mild mental ratardation)			Y (high CK and LDH)			German	
14	IVS	c.1467-2A>G	2	1		N (1pt)	N/A (1pt)		N (1pt)	N/A (1pt)			N/A	Polish	
15	Missense	c.1477C>T	9	7		N (8pts)	N/A (1pt)	Y (1pt impaired cognitive functioning and hearing loss, 1pt developmental delay, 1pt something not described)	N (4pts)	N/A (2pts)	Y (1pt high CK, 1pt high CK and LDH, 1pt high LDH 5)	N (2pts)	N/A (4pts)	European, Italian, Macedonian, Japanese, Chinese	
15	Missense	c.1502T>G	1	1			N/A			N/A			N/A	Chinese	
15	Missense	c.1514G>T	1	1			N/A			N/A			N/A	Chinese	
15	Missense	c.1567G>A	5	3	Y (1pt ocular abnormalities)	N (4pts)		Y (2pts developmental delay)	N (3pts)		Y (3pts high LDH)	N (1pt)	N/A (1pt)	Italian, Chinese	Y [4]
15	Missense	c.1573A>C	2	1			N/A	Y (1pt attention deficit disorder, 1pt hearing loss)					N/A	Unknown	
15	Missense	c.1576C>T	1	1		N			N				N/A	Japanese	
15	Missense	c.1598T>C	1	1		N		Y (umbelical hernia)			Y (high CK and LDH)			European	

Exon/ Intron	Type of Mutation	Nucleotide	Cases (num)	Families (num)	Ocular			CNS			Muscular			Nationality	Reported in LS
					Y	N	N/A	Y	N	N/A	Y	N	N/A		
15	IVS	c.1603-3G>C	1	1		N		Y (mild mental retardation)			Y (high CK and LDH)			Japanese	
17	Frameshift	c.1865delG	1	1	Y (glaucoma)					N/A			N/A	Unknown	
18	Missense	c.2039T>C	1	1	Y (cataract)				N			N		Japanese	Y [5]
18	Missense	c.2078C>T	2	1			N/A			N/A			N/A	Unknown	
20	Missense	c2206A>G	1	1		N				N/A			N/A	Japanese	
20	Missense	c.2211G>C	2	1	Y (2pts mild bilateral cortical lens opacity)				N			N		Cape Verde	
20	Missense	c.2245G>T	1	1		N			N				N/A	Korean	
20	IVS	c.2257-5G>A	1	1		N			N		Y (elevated CPK and/or LDH)			Japan	Y [6]
22	Missense	c.2396C>T	1	1		N			N				N/A	French	Y [7,8]
22	Missense	c.2435T>C	2	1	Y (1pt congenital cataract)		N/A (1pt)	Y (1pt slight delay in language development)		N/A (1pt)			N/A	Chinese	
23	Nonsense	c.2464C>T	1	1			N/A			N/A			N/A	Chinese	Y [2,4,7–10]
23	Missense	c.2486C>T	1	1		N			N			N (both CK and LDH)		Japanese	
24	Frameshift	c.2670delC	1	1			N/A			N/A			N/A	Chinese	

Table S3. List of *OCRL* pathogenic variants reported in DD2 patients. Numbering is according to the cDNA sequence (GenBank entry NM_000276.4). The A of the ATG of the Methionine initiation codon is defined as nucleotide 1. IVS: splice site variant.

Exon/ Intron	Type of Mutation	Nucleotide	Protein	Protein domain	Reference
1	IVS	c.40-14A>G	-	PH	[2]
2	Missense	c.46G>C	p.Glu16Gln	PH	[11]
3-4	Gross deletion	exons 3-4		PH	[2,12]
3	Frameshift	c.175_178delAATA	p.Asn59Alafs*14	PH	[13]
3	Gross deletion	c.187_199+449del	p.Arg63fs	PH	[14]
3	IVS	c.199+1G>A	-	PH	[13,15]
3	IVS	c.199+1G>T	-	PH	[13]
4	Nonsense	c.208G>T	p.Glu70*	PH	[13]
4	Frameshift	c.214_215delCT	p.Leu72Phefs*2	PH	[3]
4	Frameshift	c.217_218delTT	p.Leu73Aspfs*1	PH	[15,16]
5	Frameshift	c.259insT	p.Gln87Leufs*11	PH	[8]
5	Frameshift	c.260delA	p.Gln87Argfs*19	PH	[3,17–19], this paper
5	Frameshift	c.265_266insGG	p.Asp89Glyfs*18	PH	[11]
5	Nonsense	c.269G>A	p.Trp90*	PH	[20]
5	Missense	c.278A>G	p.Glu93Gly	PH	[21]
5	Frameshift	c.304_311delGAACACT G	p.Glu102Phefs*27	PH	[13,22]
5	Frameshift	c.309_310delCT	p.His103Glnfs*27	PH	This paper
5	Frameshift	c.310_313delTGTT	p.Cys104*	PH	[23–25]
5	Frameshift	c.312_315delTTTG	p.Cys104*	PH	[26]
5	Nonsense	c.314T>A	p.Leu105*	PH	[27]
6	Nonsense	c.[407T>A; 408A>G]	p.Leu136*	Linker	[11]
6	Frameshift	c.412insA	p.Thr138Asnfs*2	Linker	[17]
6	Missense	c.430G>T	p.Val144Phe	Linker	[28]
6	IVS	c.439+3A>G	-	Linker	[19], This paper
6	IVS	c.440-2A>G	-	Linker	[23]
7	Frameshift	c.487_488insAA	p.Ile164Lysfs*2	Linker	[23]
7	Nonsense	c.497C>A	p.Ser166*	Linker	[16]
7	Frameshift	c.506delA	p.Gln169Argfs*16	Linker	[8,9]
7	Frameshift	c.523delC	p.Arg175Glyfs*10	Linker	[30]
7	Frameshift	c.533delC	p.Pro178Hisfs*6	Linker	[17], this paper
7	Frameshift	c.534_543delACCTCCAC CC	p.Pro179Phefs*3	Linker	[16]
7	Frameshift	c.543delC	p.Ser183Glnfs*2	Linker	This paper
7	Frameshift	c.544delT	p.Ser183Glnfs*2	Linker	[26]
7	IVS	c.560+1G>A	-	Linker	[16]
7	IVS	c.560+1G>C	-	Linker	[19], this paper
8	Frameshift	c.614delC	p.Tyr205Tyrfs*45	5-phosphatase (stop in exon 9)	[28]
8	Frameshift	c.697delG	p.Glu233Asnfs*17	5-phosphatase (stop in exon 9)	[28]
9	Frameshift	c.723insT	p.Val244Cysfs*13	5-phosphatase	[28]
9	Missense	c.728T>C	p.Phe243Ser	5-phosphatase	[8,9,31]

9	Missense	c.821T>C	p.Ile274Thr	5-phosphatase	[2,13,17,28,32]
10	In-frame	c.833_838delGAACTG	p.Glu278_Leu279del	5-phosphatase	[30]
10	Missense	c.860T>C	p.Phe287Ser	5-phosphatase	[27]
10	Missense	c.877A>G	p.Lys293Glu	5-phosphatase	[11]
11	Missense	c.952C>T	p.Arg318Cys	5-phosphatase	[2,3,11,15,19, 22,23,33–35], this paper
11	Missense	c.953G>A	p.Arg318His	5-phosphatase	[11,13,16,28], this paper
11	Missense	c.953G>T	p.Arg318Leu	5-phosphatase	[3]
11	Missense	c.962G>A	p.Gly321Glu	5-phosphatase	[16]
12	Missense	c.1060A>C	p.Asn354His	5-phosphatase	[2]
12	Missense	c.1110C>G	p.Cys370Trp	5-phosphatase	[20]
12	Missense	c.1112T>C	p.Ile371Thr	5-phosphatase	[32]
12	Missense	c.1133C>T	p.Ala378Val	5-phosphatase	[3], this paper
12	Missense	c.1156A>G	p.Arg386Gly	5-phosphatase	[19], this paper
12	Missense	c.1177A>T	p.Ile393Phe	5-phosphatase	[15]
12	Missense	c.1196T>C	p.Phe399Ser	5-phosphatase	[32]
14	Missense	c.1419C>A	p.Phe473Leu	5-phosphatase	[28]
14	Missense	c.1430A>G	p.Tyr477Cys	5-phosphatase	[28]
14	Missense	c.1430A>T	p.Tyr477Phe	5-phosphatase	[13]
14	Missense	c.1436A>G	p.Tyr479Cys	5-phosphatase	[23]
14	IVS	c.1467-2A>G	-	5-phosphatase	[36,37]
15	Missense	c.1477C>T	p.Arg493Trp	5-phosphatase	[3,11,13,15,17,22,28]
15	Missense	c.1502T>G	p.Ile501Ser	5-phosphatase	[28]
15	Missense	c.1514G>T	p.Gly505Val	5-phosphatase	[28]
15	Missense	c.1567G>A	p.Asp523Asn	5-phosphatase	[3,26,27], this paper
15	Missense	c.1573A>C	p.Lys525Gln	5-phosphatase	[19], this paper
15	Missense	c.1576C>T	p.Pro526Ser	5-phosphatase	[21,38]
15	Missense	c.1598T>C	p.Ile533Thr	5-phosphatase	[15]
15	IVS	c.1603-3G>C	p.Val535Glyfs*6	5-phosphatase	[39]
17	Frameshift	c.1865delG	p.Gly622Alafs*22	ASH	[19], this paper
18	Missense	c.2039T>C	p.Phe680Ser	ASH	[11]
18	Missense	c.2078C>T	p.Pro693Leu	ASH	[19], this paper
20	Missense	c2206A>G	p.Lys736Gln	Rho-GAP	[13]
20	Missense	c.2211G>C	p.Glu737Asp	Rho-GAP	[27]
20	Missense	c.2245G>T	p.Ala749Ser	Rho-GAP	[8]
20	IVS	c.2257-5G>A	-	Rho-GAP	[40]
22	Missense	c.2396C>T	p.Pro799Leu	Rho-GAP	[2]
22	Missense	c.2435T>C	p.Leu812Pro	Rho-GAP	[20,41]
23	Nonsense	c.2464C>T	p.Arg822*	Rho-GAP	[28]
23	Missense	c.2486C>T	p.Pro829Leu	Rho-GAP	[11]
24	Frameshift	c.2670delC	p.Phe890Phefs*19	Rho-GAP	[28]

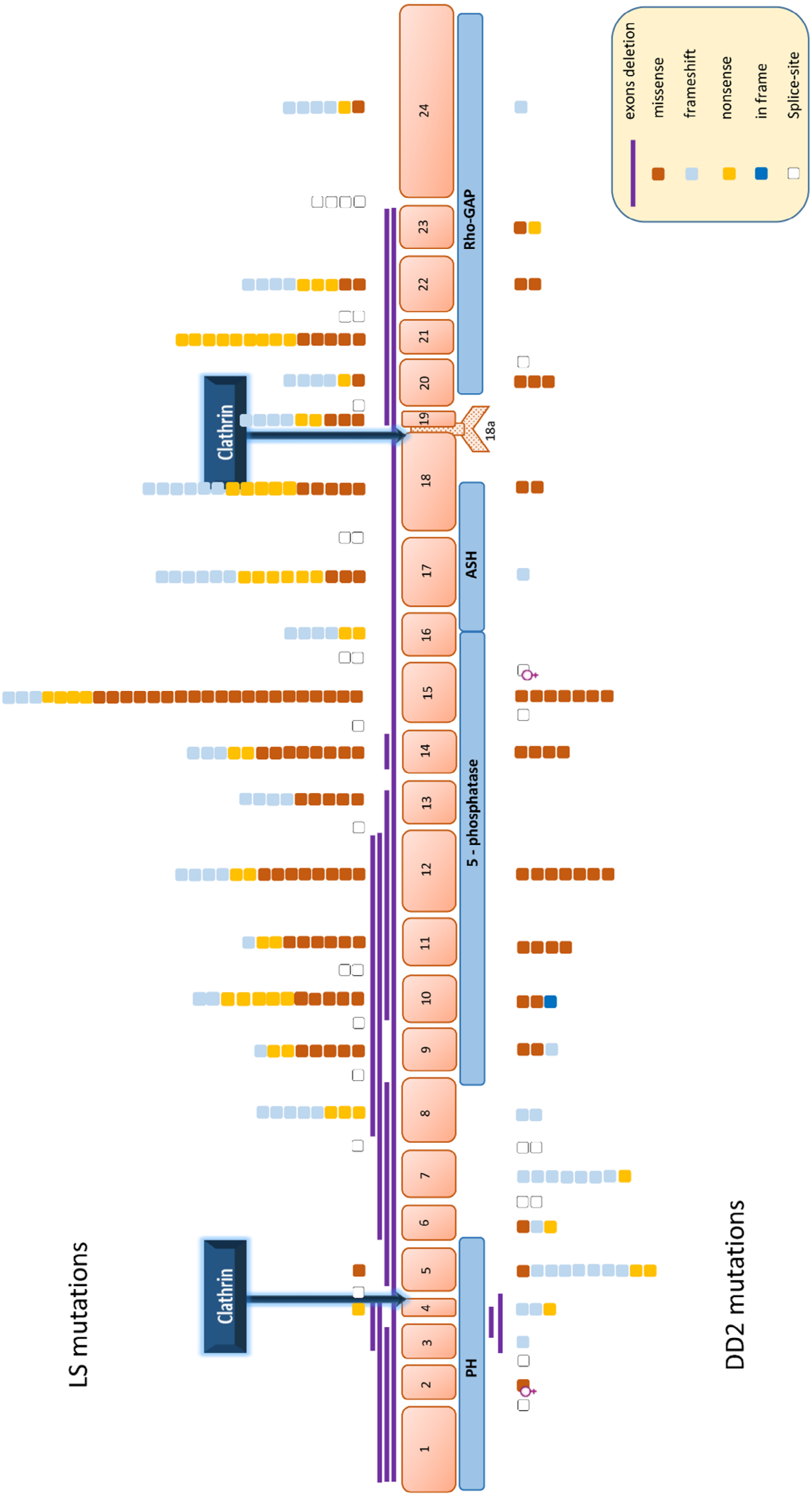

Figure S1: Genogram showing the distribution of LS and DD2 mutations along the *OCRL* gene. Upper panel shows LS mutations described in [42], lower panel shows DD2 mutations from Table S3.

References

1. Lee, B.H.; Lee, S.H.; Choi, H.J.; Kang, H.G.; Oh, S.W.; Lee, D.S.; Ha, I.S.; Choi, Y.; Cheong, H.I. Decreased Renal Uptake of (99m)Tc-DMSA in Patients with Tubular Proteinuria. *Pediatr Nephrol* **2009**, *24*, 2211–2216, doi:10.1007/s00467-009-1238-2.
2. Hichri, H.; Rendu, J.; Monnier, N.; Coutton, C.; Dorseuil, O.; Poussou, R.V.; Baujat, G.; Blanchard, A.; Nobili, F.; Ranchin, B.; et al. From Lowe Syndrome to Dent Disease: Correlations between Mutations of the OCRL1 Gene and Clinical and Biochemical Phenotypes. *Hum. Mutat.* **2011**, *32*, 379–388, doi:10.1002/humu.21391.
3. Zaniew, M.; Bökenkamp, A.; Kolbuc, M.; La Scola, C.; Baronio, F.; Niemirska, A.; Szczepanska, M.; Bürger, J.; La Manna, A.; Miklaszewska, M.; et al. Long-Term Renal Outcome in Children with OCRL Mutations: Retrospective Analysis of a Large International Cohort. *Nephrol. Dial. Transplant.* **2018**, *33*, 85–94, doi:10.1093/ndt/gfw350.
4. Recker, F.; Zaniew, M.; Böckenhauer, D.; Miglietti, N.; Bökenkamp, A.; Moczulska, A.; Rogowska-Kalisz, A.; Laube, G.; Said-Conti, V.; Kasap-Demir, B.; et al. Characterization of 28 Novel Patients Expands the Mutational and Phenotypic Spectrum of Lowe Syndrome. *Pediatr Nephrol* **2015**, *30*, 931–943, doi:10.1007/s00467-014-3013-2.
5. Sugimoto, K.; Nishi, H.; Miyazawa, T.; Fujita, S.; Okada, M.; Takemura, T. A Novel OCRL1 Mutation in a Patient with the Mild Phenotype of Lowe Syndrome. *Tohoku J Exp Med* **2014**, *232*, 163–166, doi:10.1620/tjem.232.163.
6. Nakano, E.; Yoshida, A.; Miyama, Y.; Yabuuchi, T.; Kajiho, Y.; Kanda, S.; Miura, K.; Oka, A.; Harita, Y. Incomplete Cryptic Splicing by an Intronic Mutation of OCRL in Patients with Partial Phenotypes of Lowe Syndrome. *J Hum Genet* **2020**, *65*, 831–839, doi:10.1038/s10038-020-0773-3.
7. Zaniew, M.; Bökenkamp, A.; Kolbuc, M.; La Scola, C.; Baronio, F.; Niemirska, A.; Szczepanska, M.; Bürger, J.; La Manna, A.; Miklaszewska, M.; et al. Long-Term Renal Outcome in Children with OCRL Mutations: Retrospective Analysis of a Large International Cohort. *Nephrol. Dial. Transplant.* **2018**, *33*, 85–94, doi:10.1093/ndt/gfw350.
8. Park, E.; Choi, H.J.; Lee, J.M.; Ahn, Y.H.; Kang, H.G.; Choi, Y.M.; Park, S.J.; Cho, H.Y.; Park, Y.-H.; Lee, S.J.; et al. Muscle Involvement in Dent Disease 2. *Pediatr. Nephrol.* **2014**, *29*, 2127–2132, doi:10.1007/s00467-014-2841-4.
9. Cho, H.Y.; Lee, B.H.; Choi, H.J.; Ha, I.S.; Choi, Y.; Cheong, H.I. Renal Manifestations of Dent Disease and Lowe Syndrome. *Pediatr. Nephrol.* **2008**, *23*, 243–249, doi:10.1007/s00467-007-0686-9.
10. Zheng, B.; Chen, Q.; Wang, C.; Zhou, W.; Chen, Y.; Ding, G.; Jia, Z.; Zhang, A.; Huang, S. Whole-Genome Sequencing Revealed an Interstitial Deletion Encompassing OCRL and SMARCA1 Gene in a Patient with Lowe Syndrome. *Mol Genet Genomic Med* **2019**, *7*, e876, doi:10.1002/mgg3.876.
11. Sekine, T.; Komoda, F.; Miura, K.; Takita, J.; Shimadzu, M.; Matsuyama, T.; Ashida, A.; Igarashi, T. Japanese Dent Disease Has a Wider Clinical Spectrum than Dent Disease in Europe/USA: Genetic and Clinical Studies of 86 Unrelated Patients with Low-Molecular-Weight Proteinuria. *Nephrol. Dial. Transplant.* **2014**, *29*, 376–384, doi:10.1093/ndt/gft394.
12. Duan, N.; Huang, C.; Pang, L.; Jiang, S.; Yang, W.; Li, H. Clinical Manifestation and Genetic Findings in Three Boys with Low Molecular Weight Proteinuria - Three Case Reports for Exploring Dent Disease and Fanconi Syndrome. *BMC Nephrol* **2021**, *22*, 24, doi:10.1186/s12882-020-02225-6.
13. Sakakibara, N.; Nagano, C.; Ishiko, S.; Horinouchi, T.; Yamamura, T.; Minamikawa, S.; Shima, Y.; Nakanishi, K.; Ishimori, S.; Morisada, N.; et al. Comparison of Clinical and Genetic Characteristics between Dent Disease 1 and Dent Disease 2. *Pediatr. Nephrol.* **2020**, doi:10.1007/s00467-020-04701-5.
14. Duran, D.; Jin, S.C.; DeSpenza, T.; Nelson-Williams, C.; Cogal, A.G.; Abrash, E.W.; Harris, P.C.; Lieske, J.C.; Shimshak, S.J.; Mane, S.; et al. Digenic Mutations of Human OCRL Paralogs in Dent's Disease Type 2 Associated with Chiari I Malformation. *Hum Genome Var* **2016**, *3*, 16042, doi:10.1038/hgv.2016.42.
15. Böckenhauer, D.; Bökenkamp, A.; Nuutinen, M.; Unwin, R.; Van't Hoff, W.; Sirimanna, T.; Vrljicak, K.; Ludwig, M. Novel OCRL Mutations in Patients with Dent-2 Disease. *J Pediatr Genet* **2012**, *1*, 15–23, doi:10.3233/PGE-2012-005.
16. Shrimpton, A.E.; Hoopes, R.R.; Knohl, S.J.; Hueber, P.; Reed, A.A.C.; Christie, P.T.; Igarashi, T.; Lee, P.; Lehman, A.; White, C.; et al. OCRL1 Mutations in Dent 2 Patients Suggest a Mechanism for Phenotypic Variability. *Nephron Physiol* **2009**, *112*, p27-36, doi:10.1159/000213506.

17. Utsch, B.; Bökenkamp, A.; Benz, M.R.; Besbas, N.; Dötsch, J.; Franke, I.; Fründ, S.; Gok, F.; Hoppe, B.; Karle, S.; et al. Novel OCRL1 Mutations in Patients with the Phenotype of Dent Disease. *Am. J. Kidney Dis.* **2006**, *48*, 942.e1–14, doi:10.1053/j.ajkd.2006.08.018.
18. Zhang, Y.; Fang, X.; Xu, H.; Shen, Q. Genetic Analysis of Dent's Disease and Functional Research of CLCN5 Mutations. *DNA Cell Biol.* **2017**, *36*, 1151–1158, doi:10.1089/dna.2017.3731.
19. Cogal, A.G.; Jennifer Arroyo; Shah, R.J.; Reese, K.J.; Walton, B.N.; Reynolds, L.M.; Kennedy, G.N.; Seide, B.M.; Senum, S.R.; Baum, M.; et al. Comprehensive Genetic Analysis Reveals Complexity of Monogenic Urinary Stone Disease. *Kidney Int Rep* **2021**, *submitted.*
20. Deng, H.; Zhang, Y.; Xiao, H.; Yao, Y.; Zhang, H.; Liu, X.; Su, B.; Guan, N.; Zhong, X.; Wang, S.; et al. Phenotypic Spectrum and Antialbuminuric Response to Angiotensin Converting Enzyme Inhibitor and Angiotensin Receptor Blocker Therapy in Pediatric Dent Disease. *Mol Genet Genomic Med* **2020**, e1306, doi:10.1002/mgg3.1306.
21. Suruda, C.; Tsuji, S.; Yamanouchi, S.; Kimata, T.; Huan, N.T.; Kurosawa, H.; Hirayama, Y.; Tsukaguchi, H.; Saito, A.; Kaneko, K. Decreased Urinary Excretion of the Ectodomain Form of Megalin (A-Megalin) in Children with OCRL Gene Mutations. *Pediatr Nephrol* **2017**, *32*, 621–625, doi:10.1007/s00467-016-3535-x.
22. Sekine, T.; Nozu, K.; Iyengar, R.; Fu, X.J.; Matsuo, M.; Tanaka, R.; Iijima, K.; Matsui, E.; Harita, Y.; Inatomi, J.; et al. OCRL1 Mutations in Patients with Dent Disease Phenotype in Japan. *Pediatr. Nephrol.* **2007**, *22*, 975–980, doi:10.1007/s00467-007-0454-x.
23. Hoopes, R.R.; Shrimpton, A.E.; Knohl, S.J.; Hueber, P.; Hoppe, B.; Matyus, J.; Simckes, A.; Tasic, V.; Toenshoff, B.; Suchy, S.F.; et al. Dent Disease with Mutations in OCRL1. *Am. J. Hum. Genet.* **2005**, *76*, 260–267, doi:10.1086/427887.
24. Tasic, V.; Lozanovski, V.J.; Korneti, P.; Ristoska-Bojkovska, N.; Sabolic-Avramovska, V.; Gucev, Z.; Ludwig, M. Clinical and Laboratory Features of Macedonian Children with OCRL Mutations. *Pediatr. Nephrol.* **2011**, *26*, 557–562, doi:10.1007/s00467-010-1758-9.
25. Salihu, S.; Tosheska, K.; Cekovska, S.; Tasic, V. Incidental Detection of Dent-2 Disease in an Infant with Febrile Proteinuria. *Med Princ Pract* **2018**, *27*, 392–395, doi:10.1159/000490147.
26. Bao, Y.; Suo, L.; Qian, P.; Huang, H.; Yang, Y.; Tang, J.; Zhang, M.; Li, Z.; Wang, Y.; Liang, N.; et al. Clinical and Genetic Analysis of Dent Disease with Nephrotic Range Albuminuria in Shaanxi, China. *Sci China Life Sci* **2019**, *62*, 1590–1593, doi:10.1007/s11427-018-9829-0.
27. Tosetto, E.; Addis, M.; Caridi, G.; Meloni, C.; Emma, F.; Vergine, G.; Stringini, G.; Papalia, T.; Barbano, G.; Ghiggeri, G.M.; et al. Locus Heterogeneity of Dent's Disease: OCRL1 and TMEM27 Genes in Patients with No CLCN5 Mutations. *Pediatr. Nephrol.* **2009**, *24*, 1967–1973, doi:10.1007/s00467-009-1228-4.
28. Ye, Q.; Shen, Q.; Rao, J.; Zhang, A.; Zheng, B.; Liu, X.; Shen, Y.; Chen, Z.; Wu, Y.; Hou, L.; et al. Multicenter Study of the Clinical Features and Mutation Gene Spectrum of Chinese Children with Dent Disease. *Clin. Genet.* **2020**, *97*, 407–417, doi:10.1111/cge.13663.
29. Addis, M.; Meloni, C.; Tosetto, E.; Ceol, M.; Cristofaro, R.; Melis, M.A.; Vercelloni, P.; Del Prete, D.; Marra, G.; Anglani, F. An Atypical Dent's Disease Phenotype Caused by Co-Inheritance of Mutations at CLCN5 and OCRL Genes. *Eur. J. Hum. Genet.* **2013**, *21*, 687–690, doi:10.1038/ejhg.2012.225.
30. Li, F.; Yue, Z.; Xu, T.; Chen, M.; Zhong, L.; Liu, T.; Jing, X.; Deng, J.; Hu, B.; Liu, Y.; et al. Dent Disease in Chinese Children and Findings from Heterozygous Mothers: Phenotypic Heterogeneity, Fetal Growth, and 10 Novel Mutations. *J. Pediatr.* **2016**, *174*, 204-210.e1, doi:10.1016/j.jpeds.2016.04.007.
31. Lee, B.H.; Lee, S.H.; Choi, H.J.; Kang, H.G.; Oh, S.W.; Lee, D.S.; Ha, I.S.; Choi, Y.; Cheong, H.I. Decreased Renal Uptake of (99m)Tc-DMSA in Patients with Tubular Proteinuria. *Pediatr. Nephrol.* **2009**, *24*, 2211–2216, doi:10.1007/s00467-009-1238-2.
32. Zhao, S.L.; Zhao, F.; Sha, Y.G.; Chen, Q.X.; Cheng, X.Q.; Huang, S.M. [Clinical features and genetic variants of Dent disease in 10 children]. *Zhonghua Er Ke Za Zhi* **2018**, *56*, 289–293, doi:10.3760/cma.j.issn.0578-1310.2018.04.010.
33. De Mutiis, C.; Pasini, A.; La Scola, C.; Pugliese, F.; Montini, G. Nephrotic-Range Albuminuria as the Presenting Symptom of Dent-2 Disease. *Ital J Pediatr* **2015**, *41*, 46, doi:10.1186/s13052-015-0152-4.
34. Becker-Cohen, R.; Rinat, C.; Ben-Shalom, E.; Feinstein, S.; Ivgi, H.; Frishberg, Y. Vitamin A Deficiency Associated with Urinary Retinol Binding Protein Wasting in Dent's Disease. *Pediatr Nephrol* **2012**, *27*, 1097–1102, doi:10.1007/s00467-012-2121-0.

35. Bezdíčka, M.; Langer, J.; Háček, J.; Zieg, J. Dent Disease Type 2 as a Cause of Focal Segmental Glomerulosclerosis in a 6-Year-Old Boy: A Case Report. *Front Pediatr* **2020**, *8*, 583230, doi:10.3389/fped.2020.583230.
36. Preston, R.; Naylor, R.W.; Stewart, G.; Bierzynska, A.; Saleem, M.A.; Lowe, M.; Lennon, R. A Role for OCRL in Glomerular Function and Disease. *Pediatr. Nephrol.* **2020**, *35*, 641–648, doi:10.1007/s00467-019-04317-4.
37. Bierzynska, A.; McCarthy, H.J.; Soderquest, K.; Sen, E.S.; Colby, E.; Ding, W.Y.; Nabhan, M.M.; Kerecuk, L.; Hegde, S.; Hughes, D.; et al. Genomic and Clinical Profiling of a National Nephrotic Syndrome Cohort Advocates a Precision Medicine Approach to Disease Management. *Kidney Int.* **2017**, *91*, 937–947, doi:10.1016/j.kint.2016.10.013.
38. Kaneko, K.; Hasui, M.; Hata, A.; Hata, D.; Nozu, K. Focal Segmental Glomerulosclerosis in a Boy with Dent-2 Disease. *Pediatr. Nephrol.* **2010**, *25*, 781–782, doi:10.1007/s00467-009-1362-z.
39. Okamoto, T.; Sakakibara, N.; Nozu, K.; Takahashi, T.; Hayashi, A.; Sato, Y.; Nagano, C.; Matsuo, M.; Iijima, K.; Manabe, A. Onset Mechanism of a Female Patient with Dent Disease 2. *Clin Exp Nephrol* **2020**, *24*, 946–954, doi:10.1007/s10157-020-01926-4.
40. Nakano, E.; Yoshida, A.; Miyama, Y.; Yabuuchi, T.; Kajiho, Y.; Kanda, S.; Miura, K.; Oka, A.; Harita, Y. Incomplete Cryptic Splicing by an Intronic Mutation of OCRL in Patients with Partial Phenotypes of Lowe Syndrome. *J. Hum. Genet.* **2020**, doi:10.1038/s10038-020-0773-3.
41. He, G.; Zhang, H.; Cao, S.; Xiao, H.; Yao, Y. Dent's Disease Complicated by Nephrotic Syndrome: A Case Report. *Intractable Rare Dis Res* **2016**, *5*, 297–300, doi:10.5582/irdr.2016.01058.
42. De Matteis, M.A.; Staiano, L.; Emma, F.; Devuyst, O. The 5-Phosphatase OCRL in Lowe Syndrome and Dent Disease 2. *Nat Rev Nephrol* **2017**, *13*, 455–470, doi:10.1038/nrneph.2017.83.
