# Supplementary figures and images for: Genotype Phenotype Correlation in Dent Disease 2 and Review of the Literature: OCRL Gene Pleiotropism or Extreme Phenotypic Variability of Lowe Syndrome?
Source: Genes (Basel). 2021 Oct 11;12(10):1597. doi: 10.3390/genes12101597 (PMC8535715; doi:10.3390/genes12101597)

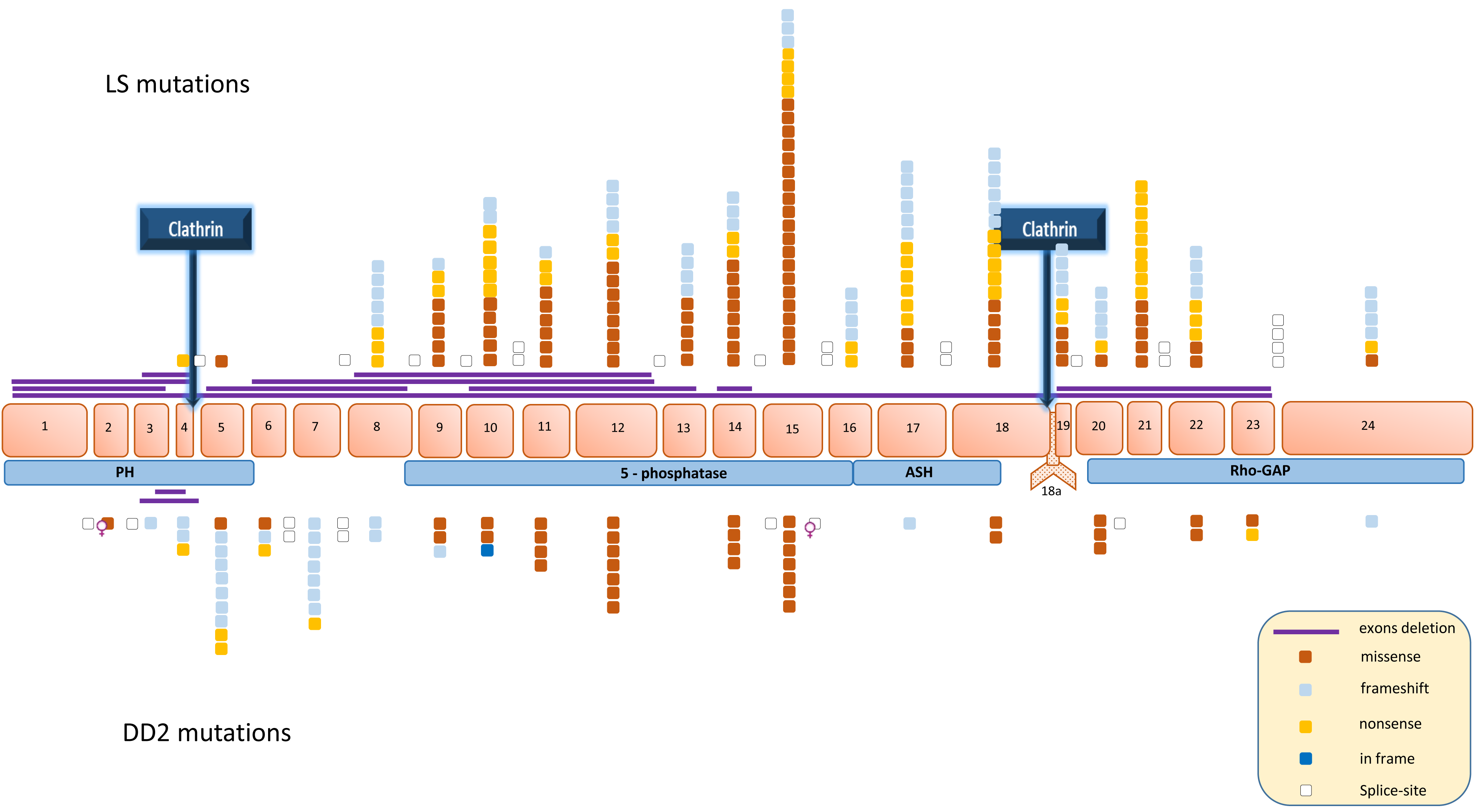

Supplement: Supplementary file 1 [file genes-12-01597-s001.zip › Figure S1.tif]
